# Clinical phenotypes from fatal cases of acute respiratory distress syndrome caused by pneumonia

**DOI:** 10.1038/s41598-021-99540-1

**Published:** 2021-10-08

**Authors:** Kazuya Ichikado, Kodai Kawamura, Takeshi Johkoh, Kiminori Fujimoto, Ayumi Shintani, Satoru Hashimoto, Yoshitomo Eguchi, Yuko Yasuda, Keisuke Anan, Naoki Shingu, Yoshihiko Sakata, Junpei Hisanaga, Tatsuya Nitawaki, Miwa Iio, Yuko Sekido, Kenta Nishiyama, Kazunori Nakamura, Moritaka Suga, Hidenori Ichiyasu, Takuro Sakagami

**Affiliations:** 1 Division of Respiratory Medicine, Saiseikai Kumamoto Hospital, 5-3-1 Chikami, Kumamoto, 861-4101 Japan; 2 Department of Radiology, Kansai Rosai Hospital, Amagasaki, Hyogo 660-8511 Japan; 3 Department of Radiology, Kurume University School of Medicine and Center for Diagnostic Imaging, Kurume University Hospital, 67 Asahi-machi, Kurume, Fukuoka 830-0011 Japan; 4 Department of Medical Statistics, Osaka City University Graduate School of Medicine, Osaka, Japan; 5 Department of Anesthesiology and Intensive Care Medicine, Kyoto Prefectural University of Medicine, Kyoto, Japan; 6 Department of Respiratory Medicine, Faculty of Life Sciences, Kumamoto University, Kumamoto 1-1-1 Honjo, Chuo-ku, Kumamoto, 860-8556 Japan

**Keywords:** Respiratory distress syndrome, Prognostic markers

## Abstract

There have been no report of objective clinical characteristics or prognostic factors that predict fatal outcome of acute respiratory distress syndrome (ARDS) since the Berlin definition was published. The aim of this study is to identify clinically available predictors that distinguish between two phenotypes of fatal ARDS due to pneumonia. In total, 104 cases of Japanese patients with pneumonia-induced ARDS were extracted from our prospectively collected database. Fatal cases were divided into early (< 7 days after diagnosis) and late (≥ 7 days) death groups, and clinical variables and prognostic factors were statistically evaluated. Of the 50 patients who died within 180 days, 18 (36%) and 32 (64%) were in the early (median 2 days, IQR [1, 5]) and late (median 16 days, IQR [13, 29]) death groups, respectively. According to multivariate regression analyses, the APACHE II score (HR 1.25, 95%CI 1.12–1.39, *p* < 0.001) and the disseminated intravascular coagulation score (HR 1.54, 95%CI 1.15–2.04, *p* = 0.003) were independent prognostic factors for early death. In contrast, late death was associated with high-resolution computed tomography (HRCT) score indicating early fibroproliferation (HR 1.28, 95%CI 1.13–1.42, *p* < 0.001) as well as the disseminated intravascular coagulation score (HR 1.24, 95%CI 1.01–1.52, *p* = 0.039). The extent of fibroproliferation on HRCT, and the APACHE II scores along with coagulation abnormalities, should be considered for use in predictive enrichment and personalized medicine for patients with ARDS due to pneumonia.

## Introduction

Acute respiratory distress syndrome (ARDS) has diverse etiologies and various clinical courses^1–3^. Two clinical phenotypes of this syndrome have recently been described, namely, phenotype 1/uninflamed and phenotype 2/hyperinflammation, as evidenced by statistical extraction independent of clinical outcomes^4,5^. Phenotypes 1 and 2 account for approximately 65% and 35% of ARDS patients, respectively. The hyperinflammation phenotype is often caused by sepsis associated with shock and metabolic acidosis, resulting in significantly higher mortality with fewer ventilator-free days and organ failure-free days compared to those with the uninflamed phenotype. Previous studies of the causes and timing of death due to ARDS in the 1980s and 1990s have reported and classified fatal cases into early and late death and emphasized primary or secondary septic syndrome as a common cause of death^6,7^. Such categorization based on timing and cause of death can likely define subgroups within the uninflamed and hyperinflammatory clinical phenotypes. Overall, because of the clinical heterogeneity of ARDS, it is critical for clinicians to understand and recognize ARDS phenotypes, along with associated prognostic factors that contribute to various outcomes, to optimize the individual treatment of patients.

Severe community-acquired pneumonia (CAP) is the most common cause of ARDS^8^*.* Additionally, ARDS due to pneumonia fulfilling the Berlin criteria concurrently satisfies the latest sepsis definition^9^. Thus, the intensity of systemic inflammation caused by pneumonia (pneumonia sepsis) in each patient possibly reflects the uninflamed and hyperinflammatory clinical phenotypes that may predict the clinical course and outcome.

Another viewpoint regarding the clinical characteristics of ARDS is based on lung pathology. For example, recent studies on the autopsy and biopsy of ARDS have reported that only half of patients who meet ARDS clinical criteria have diffuse alveolar damage (DAD) and that those with DAD had poorer outcomes than those without^10–13^. Furthermore, ARDS patients with DAD of pulmonary origin, including pneumonia, show more extensive pulmonary fibrosis at autopsy than do nonpulmonary ARDS patients, even with adjustment for the time interval from onset to death^10^. Additionally, we previously reported the clinical significance and prognostic value of high-resolution computed tomography (HRCT) for the prediction of mortality or ventilator-associated outcomes associated with secondary septic syndrome in ARDS^14,15^.

With the goal of refining therapeutic strategies against each clinical course, the objective of our study was to clarify differences in the clinical course and relevant prognostic factors among patients with different clinical outcomes including early death, late death, or survived in pneumonia-associated ARDS. We hypothesized (1) early death is positively associated with the APACHE II score and (2) late death is positively associated with the high-resolution CT score indicating the extent of fibroproliferation, associated with ventilator-associated outcomes, and these scores can predict early and late death of ARDS due to pneumonia, respectively.

## Methods

The detailed methods are described in the Online Supplementary Materials. Although this study was a retrospective, single-center study, the data were prospectively collected during an ongoing high-resolution CT (HRCT) study of patients with ARDS. Some of the study data have been published^14–21^.

### Patients

A total of 210 cases of Japanese patients with ARDS diagnosis based on the international definition of ARDS were extracted from our HRCT database encompassing from October 1, 2004, to May 31, 2017. We reviewed data to determine whether individual cases that occurred prior to the 2012 publication of the Berlin definition^4^ met those diagnostic criteria. Written informed consent was obtained from all patients or their families. The study was approved by our institutional review board (Saiseikai Kumamoto Hospital Medical Ethics Committee, Permission number 238) and was conducted in accordance with the ethical standards of the Declaration of Helsinki.

**Figure 1:**
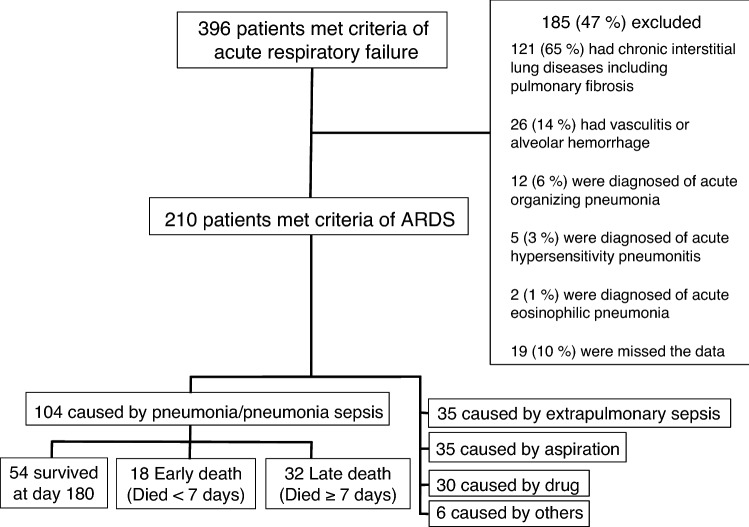
Screening and enrollment of patients with acute respiratory distress syndrome.

Patient outcomes were evaluated at 180 days after the diagnosis of ARDS and were classified into three types of prognoses; survived, early death (< 7 days after diagnosis), and late death (≥ 7 days). All patients underwent HRCT on the day of ARDS diagnosis. Exclusion criteria and the screening and enrollment flow are shown in Fig. 1.

Analysis in this study provided historical data to support a randomized, open-label multicenter phase II study evaluating the efficacy and safety of MultiStem cells [HLCM051], an allogeneic bone marrow-derived stem cell product, in patients with ARDS due to pneumonia (NCT03807804), that has been ongoing since February 2019.

### Collected data

We collected the clinical data at the diagnosis of ARDS for the patient age, sex, comorbidities, severity of ARDS, McCabe scores, acute physiology and chronic health evaluation (APACHE) II scores, sequential organ failure assessment (SOFA) scores, high-resolution CT scores (extent of fibroproliferation), high-resolution CT patterns, disseminated intravascular coagulation (DIC) scores, arterial oxygen tension (PaO_2_)/fractional inspired oxygen (FiO_2_), the extent of infiltration on chest X-ray and blood test findings. Ventilatory indexes such as PEEP, tidal volume, and peak inspiratory pressure were also collected in the groups (Table 1A, B).

**Table 1:** Background and prognosis of patients with ARDS due to pneumonia.

	Entire cohort	Comparison groups	
	All patients	Early death	The others
		(< 7 days from diagnosis)	(Late death and survivors)
**A**			
Subjects, n (%)	104 (100)	18 (17.3)	86 (82.7)
Follow-up days		2 [1, 5]	180 [24, 180]
Age	72 [65, 81]	77 [67, 83]	71.5 [65, 81]
**Sex (%)**			
Female	38 (39.5)	5 (28)	30 (34.9)
Male	66 (60.5)	13 (72)	56 (65.1)
**Comorbidity**^a^			
Chronic respiratory disease			
COPD	26 (25)	7 (39)	19 (22.1)
Others*	4 (4)	0 (0)	4 (4)
Chronic cardiovascular disease	9 (9)	2 (11)	7 (8)
Diabetes mellitus	26 (25)	4 (22)	22 (26)
Neurological disease	13 (13)	1 (5)	12 (14)
Chronic liver disease	13 (13)	1 (5)	12 (14)
Chronic renal disease	8 (8)	1 (6)	7 (8)
Immunological disease	7 (7)	2 (11)	5 (6)
Malignancy	12 (12)	1 (6)	11 (13)
**Severity of ARDS n (%)**			
Mild	20 (10)	1 (6)	7 (8)
Moderate	101 (48)	3 (17)	46 (53)
Severe	89 (42)	14 (78)	33 (38)
**Extent of infiltration on chest X-ray**			
2	13 (13)	4 (22)	9 (10)
3	53 (51)	9 (50)	44 (51)
4	38 (36)	5 (28)	33 (38)
**High-resolution CT pattern**			
Definite diffuse alveolar damage pattern	46 (56)	6 (33)	40 (46)
Possible diffuse alveolar damage pattern	18 (15)	1 (5.6)	17 (20)
Inconsistent with diffuse alveolar damage pattern	40 (29)	11 (61)	29 (34)
HRCT score	216 [185, 291]	206 [166, 253]	211 [182, 271]
**McCabe score**			
1	178 (85)	17 (94)	77 (89)
2	17 (8)	0 (0)	7 (8)
3	15 (7)	1 (6)	2 (2)
APACHE II score	22 [18, 25]	28 [24, 31]	21 [18, 25]
SOFA score	7 [5, 10]	12 [9, 14]	7 [5, 10]
PaO_2_/FiO_2_ ratio	108 [78, 157]	74 [64, 96]	110 [79, 152]
PEEP, cm	9 [8, 12]	10 [8, 13]	10 [8, 12]
Tidal volume, ml	425 [360, 500]	430 [400, 490]	422 [360, 500]
Peak inspiratory pressure, cmH2O	21 [18, 24]	22 [17.5, 24]	21 [18, 24]
**JAAM DIC score (n = 103) n (%)**			
0	3 (2.9)	0 (0.0)	3 (3)
1	18 (17.5)	1 (5.6)	17 (20)
2	26 (25.2)	1 (5.6)	25 (29)
3	16 (15.5)	4 (22.2)	12 (14)
4	17 (16.5)	4 (22.2)	13 (15)
5	12 (11.7)	4 (22.2)	8 (9)
6	5 (4.9)	1 (5.6)	4 (4)
7	6 (5.8)	3 (16.7)	3 (3)
DIC score (≥ 4)	40 (38.4)	12 (66.7)	28 (33)
Albumin (g/dL)	2.9 [2.4, 3.2]	2.8 [2.4, 3.2]	2.9 [2.4, 3.3]
WBC (/μl)	9850 [6025, 14,750]	4400 [2475, 9975]	10,050 [5150, 13,575]
CRP (mg/dl)	15.8 [9.3, 24.9]	19.9 [13.2, 32.9]	18.3 [9.7, 25.5]
LDH (IU/L)	328 [249, 461]	311 [283, 362]	274 [228, 454]
Platelet (X10^4^/μl)	18.0 [11.3, 24.7]	15.2 [7.3, 25.6]	16.8 [11.3, 22.0]

	Comparison groups		
	Survivors	Late death	
		(≥ 7 days after diagnosis)	
**B**			
Subjects, n (%)	54 (51.9)	32 (30.7)	
Follow-up days	180 [180, 180]	16 [13, 29]	
Age	73 [65, 82]	70 [65, 80]	
**Sex (%)**			
Female	20 (37.0)	10 (31.2)	
Male	34 (63.0)	22 (68.8)	
**Comorbidity**^a^			
Chronic respiratory disease			
COPD	11 (20)	8 (25)	
Others*	1 (2)	3 (9)	
Chronic cardiovascular disease	6 (11)	1 (3)	
Diabetes mellitus	9 (17)	13 (41)	
Neurological disease	10 (18)	2 (6)	
Chronic liver disease	6 (11)	6 (19)	
Chronic renal disease	4 (7)	3 (9)	
Immunological disease	2 (4)	3 (9)	
Malignancy	5 (9)	6 (19)	
**Severity of ARDS n (%)**			
Mild	3 (6)	4 (12)	
Moderate	31 (57)	15 (47)	
Severe	20 (37)	13 (41)	
**Extent of infiltration on chest X-ray**			
2	9 (17)	0 (20)	
3	31 (57)	13 (41)	
4	14 (26)	19 (59)	
**High-resolution CT pattern**			
Definite diffuse alveolar damage pattern	18 (33)	22 (67)	
Possible diffuse alveolar damage pattern	11 (21)	6 (19)	
Inconsistent with diffuse alveolar damage pattern	25 (46)	4 (12)	
HRCT score	193 [170, 221]	254 [212, 314]	
**McCabe score**			
1	51 (94)	26 (81)	
2	3 (6)	4 (13)	
3	0 (0)	2 (6)	
APACHE II score	21 [19, 25]	21 [17, 25]	
SOFA score	7 [5, 10]	7 [5, 9]	
PaO_2_/FiO_2_ ratio	111 [83, 155]	109 [78, 139]	
PEEP, cm	9 [8, 12]	10 [8, 12]	
Tidal volume, ml	420 [350, 500]	425 [375, 481]	
Peak inspiratory pressure, cmH2O	21 [18, 24]	21 [19, 24]	
**JAAM DIC score (n = 103) n (%)**			
0	1 (1.9)	2 (6.2)	
1	14 (26.4)	3 (9.4)	
2	18 (34.0)	7 (21.9)	
3	6 (11.3)	6 (18.8)	
4	6 (11.3)	7 (21.9)	
5	4 (7.5)	4 (12.5)	
6	2 (3.8)	2 (6.2)	
7	2 (3.8)	1 (3.1)	
DIC score (≥ 4)	14 (25.9)	14 (43.7)	
Albumin (g/dL)	3.0 [2.4, 3.4]	2.9 [2.5, 3.1]	
WBC (/μl)	9450 [5175, 13,025]	11,450 [5050, 14,225]	
CRP (mg/dl)	19.5 [10.7, 26.0]	15.2 [8.0, 23.5]	
LDH (IU/L)	256 [218, 337]	364 [256, 495]	
Platelet (X10^4^/μl)	17.2 [13.1, 27.1]	16.2 [8.9, 21.1]	

a. Comorbidities other than those excluded in Figure 1.

#. Others: chronic bronchiectasis, chronic bronchitis, or non-tuberculous mycobacterial infection

Continuous variables expressed as medians and interquartile ranges (IQRs).

*HRCT* high-resolution CT, *APACHE* acute physiology and chronic health evaluation, *SOFA* sequential organ failure assessment, *JAAM DIC score* Japanese Association for Acute Medicine disseminated intravascular coagulation score, *CRP* C-reactive protein, *HRCT* high-resolution computed tomography, *JAAM* Japanese Association for Acute Medicine, *DIC* disseminated intravascular coagulation, *LDH* lactate dehydrogenase, *PEEP* positive-end expiratory pressure, *WBC* white blood cell count.

### Agreement of pathogen sensitivity with initial empiric antimicrobial agents

The identification of the detected bacteria or influenza virus antigen as pathogens of pneumonia was determined in conjunction with clinical information. A bacterial pathogen was determined to be present if gram-positive or gram-negative bacteria were detected in a blood sample, sputum, endotracheal aspirates, bronchoalveolar lavage specimen; if *S. pneumoniae* and *Legionella pneumophila* was detected in urine by means of antigen detection. A viral pathogen was determined to be present if influenza virus was detected in a nasopharyngeal swab by means of PCR assay.

Microbial etiologies in three types groups of prognoses were described in Table 2. The sensitivity to initial antimicrobial agents, and prior administration of antibiotics were also recorded in three groups. Except for cases immediately diagnosed based on urine antigens such as *Streptococcus. pneumoniae* and *Legionella pneumophila* or nasopharyngeal flu antigen, empiric antibiotics were selected per Japanese Respiratory Society guidelines for the management of respiratory infections^22,23^. When necessary, antimicrobials were adjusted according to the in vitro sensitivity of cultured pathogens. Pathogen sensitivity to initial empiric antimicrobials was graded as follows: sensitive, non-sensitive, and no bacteria detected. “No bacteria detected” was assigned when no significant pathogens were cultured.

**Table 2:** Microbiological etiology and use of antipathogen agents.

	All patients	Survivors	Early death patients	Late death patients
			(< 7 days from diagnosis)	(≥ 7 days after diagnosis)
Subject, n (%)	104 (100)	54 (52)	18 (17)	32 (31)
**Pathogen of pneumonia**				
*Streptococcus pneumonia*	43 (41)	26 (48)	9 (50)	8 (25)
*Influenza virus*	8 (7)	6 (11)	1 (5)	1 (3)
*Legionella pneumophila*	4 (4)	2 (4)	1 (5)	1 (3)
*Pneumocystis jirovecii*	4 (4)	2 (4)	0 (0)	2 (6)
*Klebsiella spp.*	2 (2)	1 (2)	1 (5)	0 (0)
*Haemophilus Influenzae*	1 (1)	1 (2)	0 (0)	0 (0)
*Nocardia*	2 (2)	0 (0)	1 (5)	1 (3)
*Pseudomonas*	2 (2)	0 (0)	2 (11)	0 (0)
*E. coli*	3 (3)	2 (4)	0 (0)	1 (3)
*Cytomegalovirus*	1 (1)	0 ()	0 (0)	1 (3)
*MRSA*	1 (1)	1 (2)	0 (0)	0 (0)
No bacteria detected	33 (32)	13 (24)	3 (17)	17 (53)
**Sensitivity to initial antimicrobial agents**				
With bacteria sensitive	68 (65)	42 (78)	13 (72)	13 (41)
With bacteria not-sensitive	5 (5)	1 (2)	2 (11)	2 (6)
No bacteria detected	31 (30)	11 (20)	3 (17)	17 (53)
**Prior administration of antibiotics**				
Yes	39 (37)	15 (28)	6 (33)	18 (56)
No	65 (63)	39 (72)	12 (67)	14 (43)

*Spp* species, *MRSA* methicillin-resistant *Staphylococcus aureus.*

### Assessment of chest radiography and HRCT findings

The extent of chest radiography lung infiltration was scored by the Murray score^24^, as follows: 1-quadrant, 2-quadrants; 3-quadrants; and 4-quadrants. All patients underwent helical HRCT of the chest on the day of ARDS diagnosis using multidetector-row CT (MDCT). This study evaluated single HRCT scans acquired on the day of ARDS diagnosis because of the difficulty in obtaining sequential scans in patients receiving positive-pressure ventilation. The HRCT scans were independently evaluated by two experienced chest radiologists (T.J. and K.F.) who were unaware of the patients’ clinical condition.

### Evaluation of HRCT patterns and HRCT score

The DAD pattern on HRCT is characterized by patchy ground-glass attenuation and/or air-space consolidation associated with bronchial dilation, reticular opacities, and cystic changes depending on the pathologic fibroproliferative phase of DAD according to the most recent international multidisciplinary consensus statement of idiopathic interstitial pneumonias^24^. Each patient’s HRCT scan was assigned one of three patterns (definite DAD pattern, possible DAD pattern, and inconsistent with DAD pattern), consistent with the international guideline (Supplement Figure 2). In addition, HRCT findings were graded a score of 1–6 based on the classification system correlating with previously described pathology^25^. The scoring system has been reported^14,15,25^.

### Assignment of ventilator-associated outcomes

We reported that early fibroproliferation on HRCT scans at diagnosis of ARDS increased risk of prolonged ventilation, ventilator-associated pneumonia, and air leak syndrome, resulting in secondary sepsis syndrome^15^. To evaluate the relation between the HRCT scores and these ventilator-associated outcomes in patients with ARDS due to pneumonia, these outcomes were recorded; whether the patient was weaned from the ventilator within 28 days after the diagnosis; whether air leak syndrome defined as any pneumothorax, pneumomediastinum or subcutaneous emphysema was noted as present or absent on regular chest radiographs; whether diagnoses of culture-confirmed ventilator-associated tracheobronchitis or pneumonia requiring newly prescribed antibiotics were done.

### Evaluation of coagulation and fibrinolytic abnormalities

Coagulation and fibrinolytic abnormalities at ARDS diagnosis were assessed by the disseminated intravascular coagulation (DIC) score criteria of the Japanese Association of Acute Medicine (JAAM)^26^.

### Statistical analysis

Continuous variables expressed as medians and interquartile ranges (IQRs), and categorical variables in each three group (survived, early death and late death) are shown. The interobserver variation of the presence/absence of the HRCT findings was analyzed using the weighted *kappa* statistic and classified as follows: poor (*kappa* = 0–0.20), fair (*kappa* = 0.21–0.40), moderate (*kappa* = 0.41–0.60), substantial (*kappa* = 0.61–0.80), and almost perfect (*kappa* = 0.81–1.00). Interobserver variation regarding the extent of the HRCT findings was assessed by Spearman’s rank correlation coefficient, and the HRCT scores assigned by the two independent observers were compared using the Bland–Altman method (Supplementary Figure 3). If the HRCT patterns or the HRCT scores did not agree between the two radiologists, one of the patterns or the scores was adopted by consensus. Of the HRCT scores which were semiquantitative markers of fibroproliferation, and the three patterns on HRCT scan (definite DAD pattern, possible DAD pattern, and inconsistent with DAD pattern), which were qualitative indicators, the HRCT scores were adopted for multivariate analysis because of the results of our previous studies^14–21^. From our published studies^14–21^ as described in our study hypothesis, we built the model for multivariate analysis using age, the APACHE II score, the HRCT score, and the DIC score.

To evaluate whether the APACHE II score would be a prognostic factor of early death (< 7-day mortality), univariate and multivariate analyses using Cox proportional hazard models were performed among the two groups; early death group versus late death group plus survived one. Similarly, to assess whether the HRCT score would be predictive factors for the late death (from day 7 to day 180), univariate and multivariate analyses were done between the two groups; late death group versus survived one. We employed receiver operating characteristic (ROC) curve analysis to examine the sensitivity, specificity and predictive values of the APACHE II score and HRCT score and identified the best cutoff value for each with Youden’s index. The relation among the HRCT scores and ventilator-related outcomes (weaning of ventilator within 28 days, air-leak syndrome, and ventilator-associated infection) were evaluated using Logistic regression analyses after adjusting age and severity of ARDS in the late death groups and survived one. For all analyses, a p-value less than 0.05 was considered to indicate a statistically significant difference.

## Results

Of the 210 cases, 104 with a diagnosis of ARDS due to pneumonia were extracted from our database (Fig. 1). Patient demographics and severity characteristics are shown in Table 1. The results of microbial etiology, antimicrobial treatment and sensitivity to initial antimicrobial agents are shown in Table 2.

### The timing and causes of death

**Figure 2:**
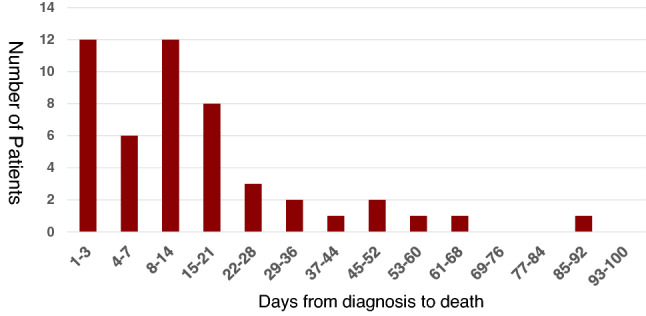
Correlation between the number of patients who died and days from diagnosis to death. Fifty (48%) of 104 patients in our series died during the 180-day study period, and 82% (41 deaths) of all deaths occurred within the first 28 days. Eighteen (36%) of the 50 nonsurvivors died within six days (< 7 days) of diagnosis (median, 2 days, IQR [1, 5]; early death group). The other 32 patients (64%) died after day 7 (≥ 7 days) (median, 16 days, IQR [13, 29]; late death group).

The distribution of days from diagnosis to death in this study is depicted in Fig. 2. Fifty of 104 patients (48%) died during the 180-day study period, with 41 (82% of deaths) occurring within the first 28 days. Eighteen (36%) died less than 7 days following diagnosis (median, 2 days, IQR [1, 5]; early death group), and almost all deaths (88%) were due to sepsis syndrome associated with multiple organ failure or shock. Of 32 patients (64%) who died after ≥ 7 days (median, 16 days, IQR [13, 29]; late death group), 22 (68%) died of sepsis syndrome and 10 (32%) of irreversible respiratory failure.

### Differences of clinical characteristics

There were distinct clinical features in the early and late death groups. The demographics and clinical characteristics of the patients in the three outcome groups (survivors, early deaths and late deaths) are summarized in Table 1A, B. In comparison among the groups (the early death group vs. the others; the late death group plus survived one) (Table 1A), the early death group had a more severe general condition, as reflected by APACHE II score (Early; median, 28 IQR [24, 31] vs. Others; median, 21 IQR [18, 25]), and more severe ARDS itself (Early; PaO_2_/FiO_2_ median, 74 IQR [64, 96] vs. Others: PaO_2_/FiO_2_ median, 110 IQR [79, 152]) than those in the others group. The early death group also had higher sequential organ failure assessment (SOFA) (Early; median, 12 IQR [9, 14] vs. Others; median, 7 IQR [5, 10]) and the higher percentage of overt disseminated intravascular coagulation (DIC) with a DIC score of 4 or higher (Early; 67% vs. Others; 33%) than the others group. Conversely, compared between the late death group and survived one (Table 1B), the severity of fibroproliferation, as evidenced by the extent of areas with traction bronchiectasis (contributing to high HRCT scores), was higher in the late death group (median, 254; IQR [212, 314]) than in the survived one (median, 193; IQR [170, 221]).

Pneumonia microbial pathogens were identified in 70% of patients (Table 2), and pathogens were identified most frequently in the early death group (83%) and least frequently in the late death group (47%). Prior administration of antimicrobial agents at referring hospitals was significantly more frequent common in the late death group (56%) than in the early death group (33%) and survivors (28%). Similarly, cases where no bacteria were detected in culture, were more frequent in the late death group (53%) than in the other two groups. *S. pneumonia* was the most frequent pathogen isolated (41% of all cases) and tended to be the major bacterium causing early death (50%).

### Difference in high-resolution CT features

There was substantial agreement (weighted kappa 0.78) between the 2 experts’ evaluations of HRCT findings. The agreement between the 2 observers was also good in assessing the extent of the lung field lesions (rs = 0.83, *p* < 0.001). The consistency of HRCT scores between the 2 observers is shown in Bland–Altman plots, with 95% limits of agreement (Supplement Figure 2). For all patients, HRCT patterns were assessed as definite (56%), possible (15%), or inconsistent with DAD (29%). A definite DAD pattern was most frequent (67%) in the late death group. Nevertheless, inconsistency in the DAD pattern was observed in 61% of the patients who died early.

### Prognostic implications

#### Predictor of early death

In comparison among the groups (the early death vs. the late death plus survived one), the APACHE II score (HR 1.25, 95% CI 1.12–1.39, *p* < 0.001) and the DIC score (HR 1.54, 95%CI 1.15–2.04, *p* = 0.003) were independently associated with early death (< 7 days) based on multivariate regression analysis (Table 3). In addition, ROC curve analysis showed that an APACHE II score of 27 was appropriate for the prediction of early death, with 67% sensitivity and 84% specificity (AUC, 0.79; 95%CI, 0.68–0.91) (Supplementary Figure 1).

**Table 3:** Univariate and multivariate analyses of mortality.

Variable	Univariate for early death (< 7 days)			Multivariate for early death (< 7 days)			Univariate for late death (≥ 7 days)			Multivariate for late death (≥ 7 days)		
	HR	95% CI	*P* value	HR	95% CI	*P* value	HR	95% CI	*P* value	HR	95% CI	*P* value
Age	1.02	0.98–1.07	0.229	1.04	0.98–1.09	0.119	1	0.97–1.03	0.989	1.01	0.98–1.05	0.37
Sex	1.32	0.47–3.71	0.593				1.3	0.61–2.70	0.491			
**Comorbidity**												
Diabetes mellitus	0.79	0.26–2.4	0.686				2.45	1.21–4.98	0.01			
Neurological disease	0.37	0.05–2.84	0.345				0.37	0.09–1.54	0.172			
COPD	1.98	0.77–5.12	0.156				1.29	0.58–2.88	0.526			
Other chronic respiratory disease	1.68	0.52–5.41	0.384				2.96	0.89–9.76	0.075			
Cardiovascular disease	1.4	0.32–6.09	0.652				0.29	0.04–2.15	0.228			
Chronic liver disease	0.38	0.05–2.88	0.361				1.36	0.54–3.24	0.523			
Chronic renal disease	0.66	0.08–4.98	0.69				0.97	0.35–2.70	0.956			
Malignancy ex	0.43	0.05–3.25	0.415				1.15	0.35–3.77	0.818			
Immunological disease	1.84	0.424–8.03	0.413				2.13	0.64–7.00	0.213			
McCabe	0.9	0.26–3.07	0.878				1.95	1.02–3.73	0.043			
APACH II score	1.23	1.12–1.35	< 0.001	1.25	1.12–1.39	< 0.001	0.968	0.90–1.03	0.341	0.98	0.90–1.05	0.537
SOFA score	1.29	1.13–1.48	< 0.001				0.96	0.87–1.07	0.55			
PaO_2_/FiO_2_ ratio	0.98	0.97–0.99	0.02				0.99	0.99–1.00	0.982			
Severity of the Berlin definition	3.34	1.26–8.87	0.01				0.94	0.53–1.68	0.844			
Extent of infiltration on chest X-ray	0.56	0.28–1.13	0.106				2.89	1.53–5.46	0.001			
JAAM DIC score	1.46	1.14–1.86	0.002	1.54	1.15–2.04	0.003	1.15	0.95–1.38	0.139	1.24	1.01–1.52	0.039
HRCT score*	0.99	0.98–1.00	0.44	1.13	0.90–1.42	0.286	1.25	1.05–1.38	< 0.001	1.28	1.13–1.42	< 0.001
HRCT score ≥ 211	0.94	0.37–2.37	0.89				5.93	2.43–14.5	< 0.001			
**HRCT pattern**												
Definite diffuse alveolar damage	0.43	0.16–1.171	0.101				5.34	1.84–15.53	0.002			
Possible diffuse alveolar damage	0.18	0.023–1.393	0.101				2.84	0.80–10.05	0.106			
Inconsistent with diffuse alveolar damage^a^	Reference						Reference					
Prior antibiotics^b^	0.82	0.31–2.18	0.691				2.57	1.28–5.19	0.008			
Sensitivity of initial antibiotics^c^	0.45	0.10–1.99	0.292				0.21	0.05–0.95	0.043			

Univariate and multivariate analyses for the early death were performed among the groups (the early death group (n = 18) vs. the late death group (n = 32) plus survived one (n = 54)).

Univariate and multivariate analyses for the late death were performed between the late death group (n = 32) and survived one (n = 54).

*SOFA* sequential organ failure assessment, *JAAM DIC score* Japanese Association of Acute Medicine disseminated intravascular coagulation score, *HRCT score* high-resolution computed tomography score.

*The hazard ratio of the HRCT score is expressed as mortality change per 10% increase in area of attenuation with traction bronchiectasis on high-resolution CT.

^a^Inconsistent with diffuse alveolar damage pattern was used as a reference to calculate the relative risk of the other two patterns.

^b^Prior antibiotics; No. of cases, Early death: 6 (33%), Late death: 18 (56%), Survived: 15 (28%).

^c^Sensitivity of initial antibiotics; Number of cases where no bacteria were detected in culture, Early death: 3(17%), Late death: 17(53%), Survived: 11 (20%).

^b,c^Prior antibiotics and sensitivity of initial antibiotics were excluded from multivariate analysis because of the uncertainty and the large number of cases where organism was not detected in culture.

#### Predictor of late death

**Figure 3:**
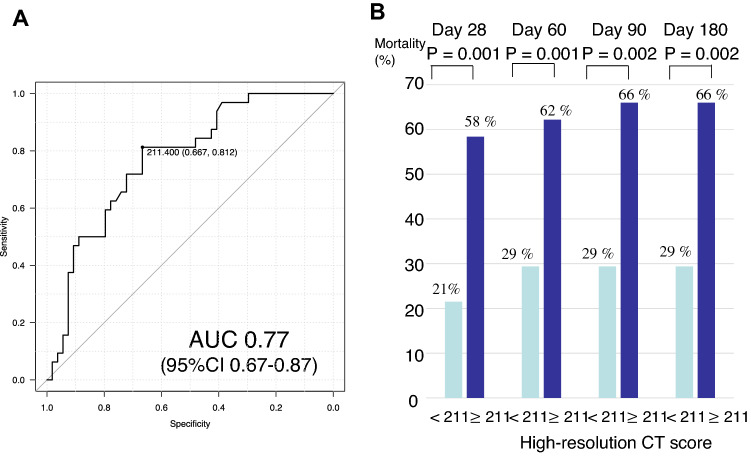
(**A**) Receiver operator characteristic (ROC) curve of the predictive value of the high-resolution CT score for late death (≥ 7 days from diagnosis). The ROC curve yielded an optimal cutoff value of the HRCT score of 211, which was determined by the Youden Index for the prediction of death from day 7 to day 180, with 81% sensitivity and 67% specificity (AUC, 0.771; 95%CI, 0.671–0.872). (**B**) Each mortality rate was compared between the optimal cutoff value of the high-resolution CT score. The mortality rate of patients with HRCT scores less than 211 ranged between 20 and 30% at any time point; the rate of those with an HRCT score of 211 or greater ranged between 58 and 66%. Statistical differences were noted between the 2 groups at all time points.

Multivariate regression analysis after adjusting for characteristics showed that the HRCT score (HR 1.28, 95%CI 1.13–1.42, *p* < 0.001) as well as the DIC score (HR 1.24, 95%CI 1.01–1.52, *p* = 0.039) was independently associated with late death (Table 3). The ROC curve yielded an optimal cutoff value of 211 for the HRCT score, which was determined by the Youden Index to predict death from day 7 to day 180, with 81% sensitivity and 67% specificity (AUC, 0.77; 95%CI, 0.67–0.87) (Fig. 3A). The mortality rate of patients with HRCT scores lower than 211 was in the 20% range for each time point; those with an HRCT score of 211 or higher had a mortality rate in the 60% range (Fig. 3B).

#### Predictors of ventilator-associated outcomes

In our adjusted analysis, the HRCT score was independently associated with ventilator weaning within 28 days after diagnosis (OR 0.98, 95%CI 0.97–0.99, *p* < 0.001), the onset of air-leak syndrome (OR 1.02, 95%CI 1.00–1.04, *p* = 0.02), and the antibiotic-treated ventilator-associated infection (OR 1.01, 95%CI 1.0–1.02, *p* = 0.029).

## Discussion

We demonstrate here the distinct differences and prognostic factors in the early and the late death groups and clarified two clinical phenotypes among fatal cases in our patients with ARDS due to pneumonia. Early death accounted for 36% of all deaths, and these patients experienced a more severe general condition caused by the so-called “cytokine storm”, which was evidenced by higher APACHE II, SOFA, and DIC scores as well as higher disease severity despite less extensive radiological features. Conversely, patients who died late accounted for 64% of deaths; these patients were characterized by more extensive radiographic infiltration and more severe lung fibroproliferation on HRCT scans and typically experienced prolonged mechanical ventilation followed by secondary multiple organ failure. We also report here for the first time the radiological differences as well as other clinical differences in the two groups of fatal cases. Similar to our study, previous studies of the causes and timing of death among ARDS cases from the 1980s and 1990s showed that fatal cases could be classified into early (< 72 h after diagnosis) and late (> 72 h) death, emphasizing the common cause of death as sepsis syndrome^6,7^. However, our data suggest that different processes are involved in sepsis syndrome-induced death early and late. In contrast, approximately 90% of patients who died early succumbed to primary sepsis syndrome, which was also the cause of ARDS itself, whereas 60% of those who died late had secondary sepsis syndrome following prolonged mechanical ventilation. Recently, ARDS has been classified into two clinical phenotypes by using latent class analysis: uninflamed (phenotype 1) and hyperinflammation (phenotype 2)^4,5^. Although personalized medicine for ARDS would be expected according to the phenotype^5^, two phenotypes from among our fatal cases may be subgroups of phenotypes 1 and 2.

We report that evidence of early fibroproliferation based on HRCT scans at diagnosis is independently associated with ventilator-associated outcomes and subsequent multiple organ failure, as well as refractory respiratory failure^14,15^. In a prospective cohort study evaluating 159 autopsied lungs from ARDS patients, Thille et al.^10^ described that early fibroproliferation occurred within one week and fibrosis one week later. Interestingly, fibrosis was found to be more frequent in ARDS of pulmonary origin than in ARDS of other origins. As ARDS in our study was caused by pneumonia, early progression of lung fibroproliferation evaluated by HRCT score was the most relevant risk factor for late death and was considered the most crucial.

A new frontier in ARDS clinical trials involving phenotyping of patients before randomization has been proposed. Personalized mechanical ventilation tailored to the type of CT pattern of the patient (focal or nonfocal) has already been reported in this field^27^. This study was undertaken to plan the study design for a randomized, open-label multicenter phase II study to evaluate the efficacy and safety of MultiStem cells [HLCM051], an allogeneic bone marrow-derived stem cell product, in patients with ARDS due to pneumonia (NCT03807804). Using the cutoff value of APACHE II score ≥ 27, patients who are likely to die of severe systemic organ failure in a few days without confirming the effect of the investigational new drug would be excluded. On the other hand, patients who are at a high risk of progressive pulmonary fibroproliferation associated with secondary septic syndrome and could hardly be rescued by any conventional treatment were identified by using HRCT score ≥ 211.

Coagulation and fibrinolytic abnormalities that result in DIC and excessive systemic inflammation lead to multiple organ failure^28^. Although these abnormalities have to date garnered relatively little attention among ARDS researchers^18,20^, there is emerging concern about these abnormalities, as coronavirus disease 2019 (COVID-19) is reported to evoke prominent coagulopathy associated with an increased risk of death^29^. Because the DIC score was an independent prognostic factor both for early and late death, a new assessment of these abnormalities may be necessary in patients with ARDS caused by other pneumonia pathogens as well as severe acute respiratory syndrome due to coronavirus 2 (SARS-CoV-2).

Recent studies have indicated that approximately one-half of patients with ARDS who meet the Berlin definition exhibit DAD and that the prognosis of those with DAD is inferior to that of those without DAD^12,13^. In our study, the definite DAD pattern was most frequently observed in the late death group, whereas the inconsistent DAD pattern significantly was predominant in the early death group. Based on 36 autopsy cases of ARDS due to pneumonia, Sarmiento et al.^30^ reported that pathological alterations in DAD were detected in less than 50% of patients who died within six days. The distinct difference in HRCT findings between the early- and late-death groups may reflect a difference in the underlying pathology. In general, it is critical to identify DAD without using invasive procedures^31^. Further research is needed to evaluate the relationship between the HRCT patterns and prognosis.

In a study of 432 patients requiring mechanical ventilation for severe CAP, including 125 (29%) cases of ARDS, multivariate logistic regression analyses showed previous antibiotic use and inadequate antibiotic therapy to be independently associated with 30-day mortality^8^. In our study, “previous antibiotic use” and “no bacteria detected in culture” were more frequent in the late death group. It has been reported that a duration of antibiotic therapy of over 24 h may lead to reduced sensitivity in the detection of significant pathogens^32^. Hence, the longer use of broad-spectrum antibiotics without de-escalation according to the sensitivity of cultured pathogens might have resulted in antibiotic-treated ventilator-associated infection, which was most often observed in the late death group.

Our study has several limitations. First, it was a retrospective evaluation using a prospectively collected dataset. Compared with a typical retrospective design, however, our study is strengthened by the use of a prospectively enrolled cohort including prospective identification of acute respiratory failure as suspected ARDS. Second, this study included a relatively small number of patients and was conducted at a single center, which necessitates cautious extrapolation of the findings to other settings. Although we have previously reported the critical utility of HRCT findings and scoring and the prognostic value of the DIC score for the care of ARDS patients^19,20^, CT findings and coagulative and fibrinolytic abnormalities have only recently gained more widespread consideration during the current pandemic of COVID-19-associated severe acute respiratory syndrome. Third, the long period of recruitment (14 years) may have affected patient care. Despite introducing advances in ventilatory management^33^ and other supportive care over time, fundamental management, including a lung-protective ventilation strategy, did not change during our cohort. Finally, except for influenza viruses, respiratory viruses were not routinely identified because of limitations in diagnostic techniques during the study period. In cases for which no significant pathogen was identified, these patients might have been infected with other respiratory viruses, and even if these viruses were involved in the disease, only supportive care could be taken in addition to lung-protective ventilation.

## Conclusions

There are distinct differences in the clinical course and relevant prognostic factors among patients with different clinical outcomes including early death, late death, or survived in pneumonia-associated ARDS. Early death is positively associated with the APACHE II score indicating of the severity of the general condition characterized by cytokine storms, and late death is positively associated with the high-resolution CT score suggesting the early fibroproliferation, associated with higher risk of ventilator related outcomes. These scores can predict early and late death of ARDS due to pneumonia, respectively.

Systemic severity, the extent of early fibroproliferation on HRCT scans, and coagulation abnormalities should be taken into consideration in personalized medicine for ARDS caused by pneumonia.

## Supplementary Information

Supplementary Figure 1.

Supplementary Figure 2.

Supplementary Figure 3.

Supplementary Information.

## Data Availability

Yes. The datasets used and/or analyzed during the current study are available from the corresponding author on reasonable request.

## Abbreviations

ARDS: Acute respiratory distress syndrome
DAD: Diffuse alveolar damage
APACHE: Acute physiology and chronic health evaluation
HRCT: High-resolution computed tomography
IQR: Interquartile range
PBW: Predicted body weight
PEEP: Positive end-expiratory pressure
PIP: Peak inspiratory pressure
SOFA: Sequential organ failure assessment
V_T_: Tidal volume
