# Supplementary figures and images for: Clinical phenotypes from fatal cases of acute respiratory distress syndrome caused by pneumonia
Source: Sci Rep. 2021 Oct 8;11:20051. doi: 10.1038/s41598-021-99540-1 (PMC8501115; doi:10.1038/s41598-021-99540-1)

Supplemented Figure 1.

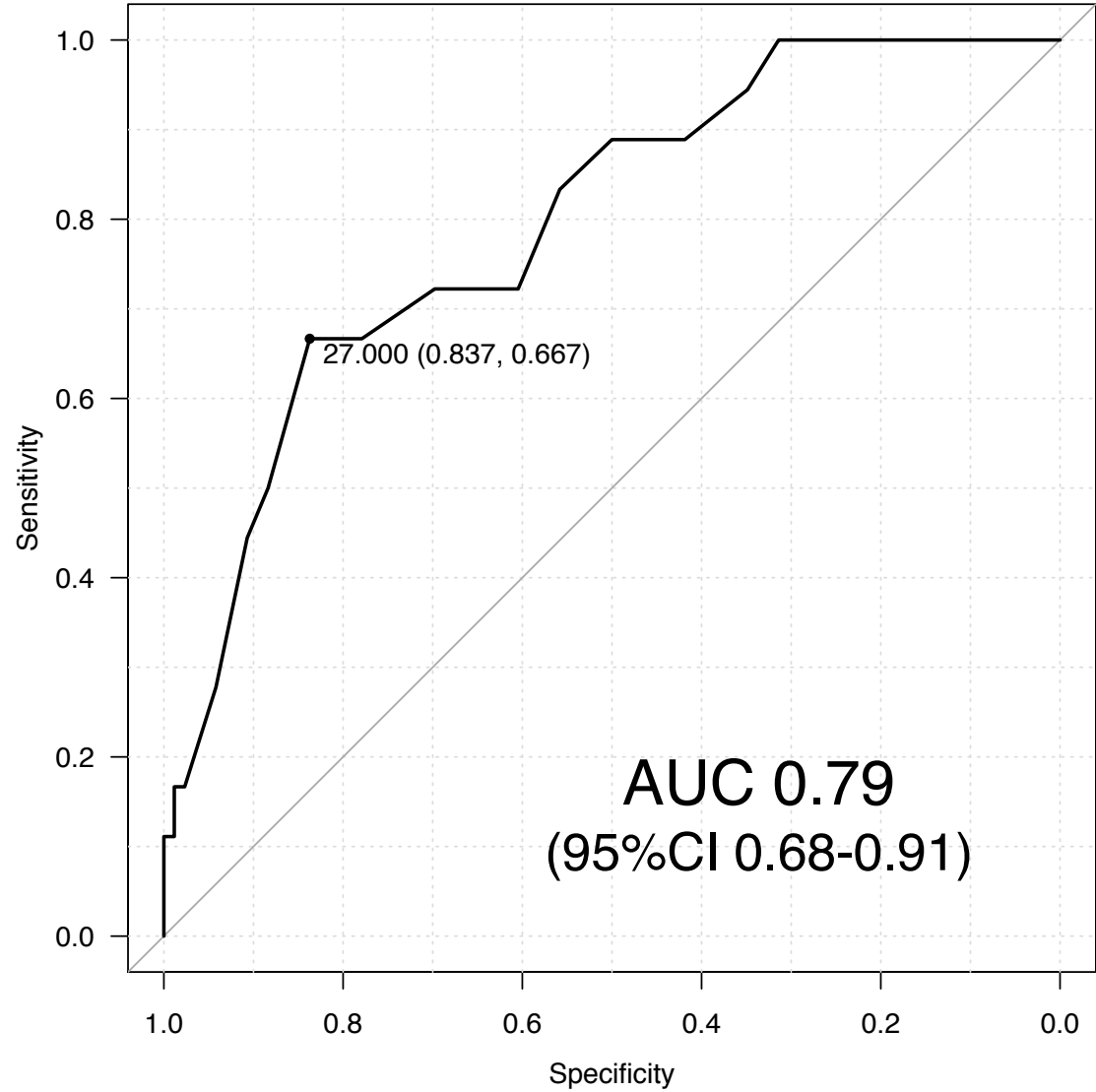

Supplement: Supplementary file 1 — Supplementary Figure 1. [file 41598_2021_99540_MOESM1_ESM.pdf]

Supplemented Figure 3: Bland-Altman plots

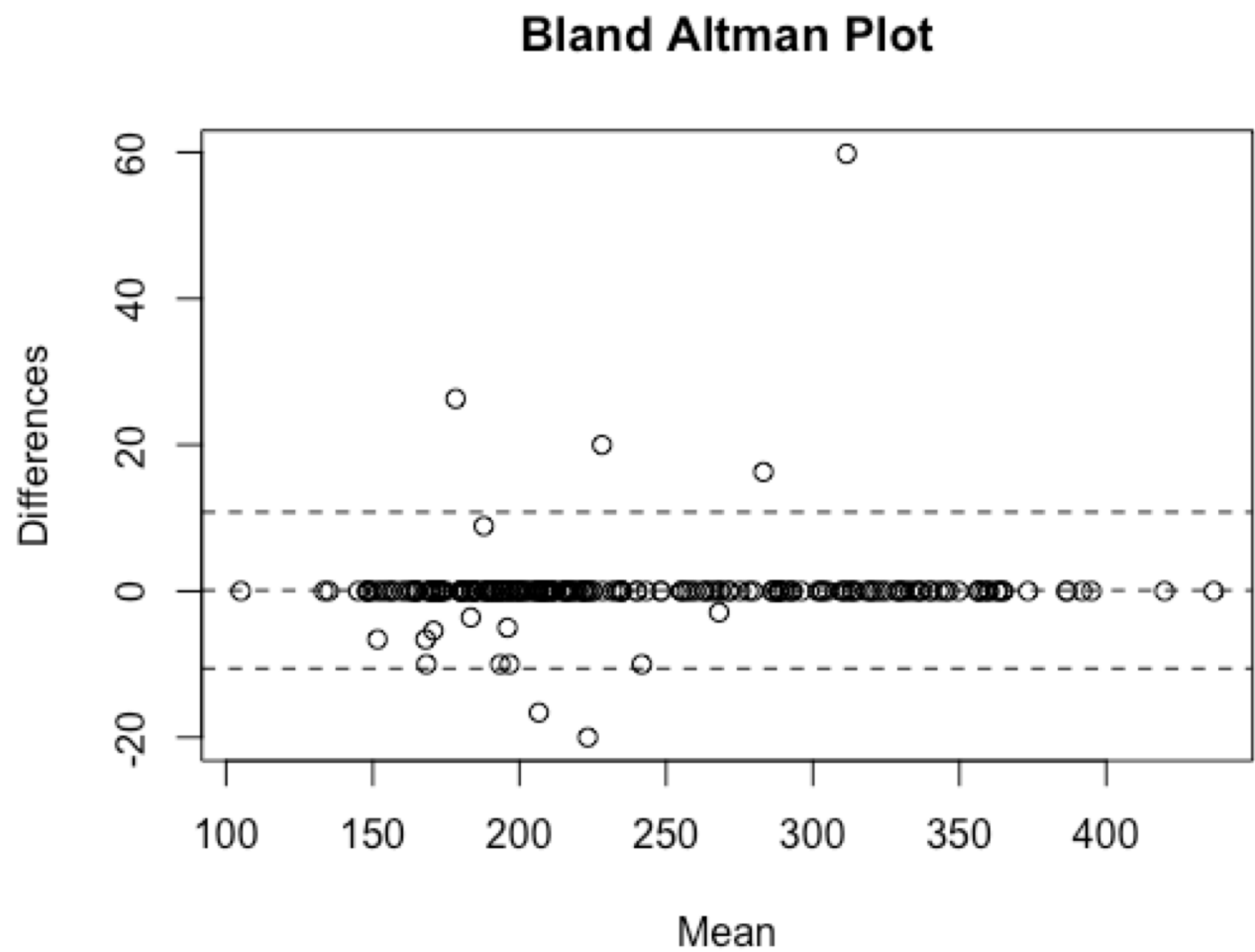

Supplement: Supplementary file 3 — Supplementary Figure 3. [file 41598_2021_99540_MOESM3_ESM.pdf]
