# Supplementary material for: Clinical phenotypes from fatal cases of acute respiratory distress syndrome caused by pneumonia
Source: Sci Rep. 2021 Oct 8;11:20051. doi: 10.1038/s41598-021-99540-1 (PMC8501115; doi:10.1038/s41598-021-99540-1)

Supplemented Figure 2: high-resolution CT patterns

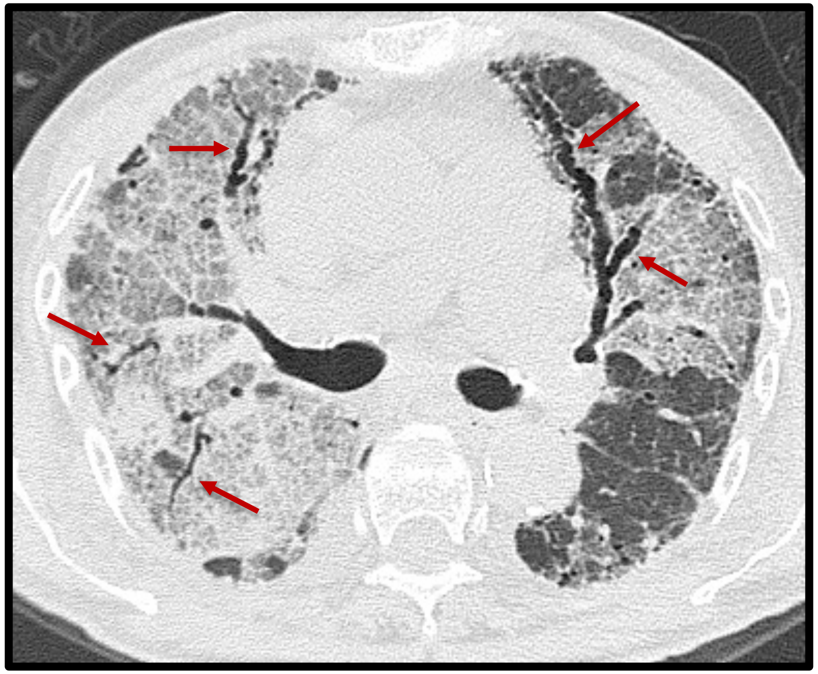

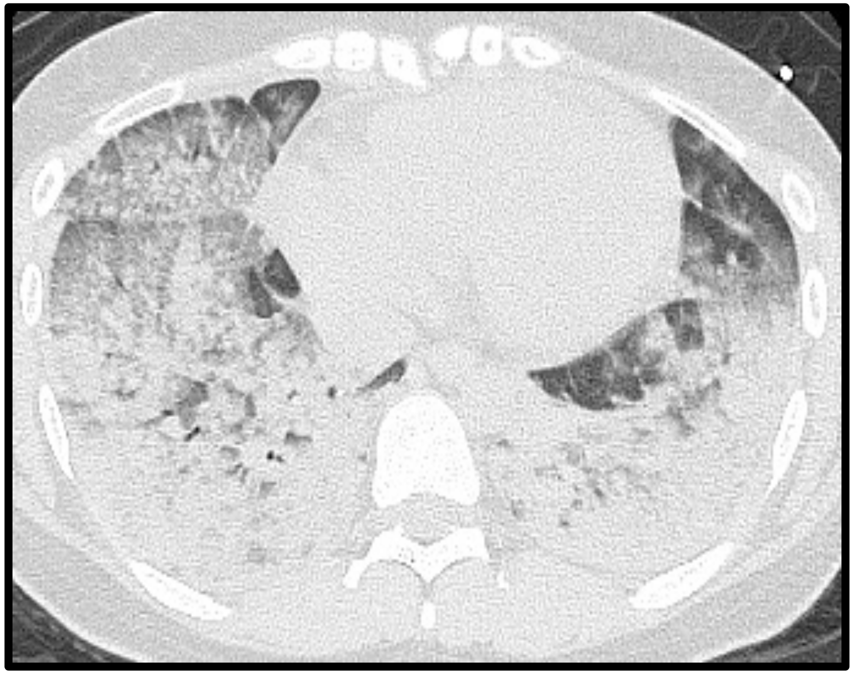

Definite Diffuse Alveolar Damage pattern	Possible Diffuse Alveolar Damage pattern
	Inconsistent with Diffuse Alveolar Damage pattern

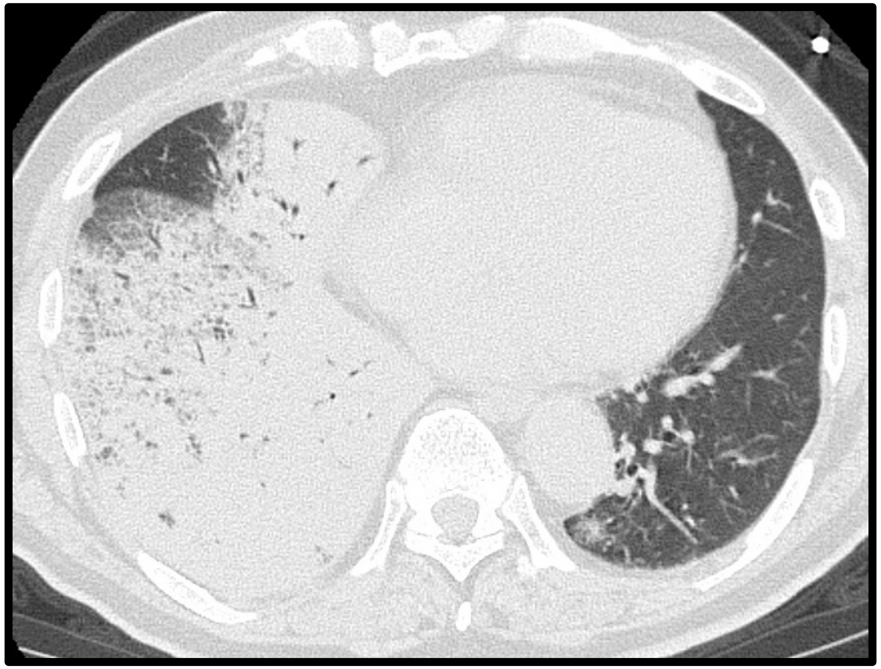

Supplement: Supplementary file 2 — Supplementary Figure 2. [file 41598_2021_99540_MOESM2_ESM.pdf]
