# Supplementary material for: Clinical phenotypes from fatal cases of acute respiratory distress syndrome caused by pneumonia
Source: Sci Rep. 2021 Oct 8;11:20051. doi: 10.1038/s41598-021-99540-1 (PMC8501115; doi:10.1038/s41598-021-99540-1)
Supplement: Supplementary file 4 — Supplementary Information. [file 41598_2021_99540_MOESM4_ESM.pdf]

Online Data Supplement

Clinical Phenotypes from Fatal Cases of Acute Respiratory Distress Syndrome Caused by Pneumonia:

Kazuya Ichikado, MD, PhD[1],
Kodai Kawamura, MD, PhD[1], Takeshi Johkoh, MD, PhD[2], Kiminori Fujimoto, MD, PhD[3], Ayumi Shintani, PhD[4], Satoru Hashimoto, MD[5], Yoshitomo Eguchi, MD[1], Yuko Yasuda, MD[1], Keisuke Anan, MD[1], Naoki Shingu, MD[1], Yoshihiko Sakata, MD[1], Junpei Hisanaga, MD[1], Tatsuya Nitawaki, MD[1], Miwa Iio, MD[1], Yuko Sekido, MD[1], Kenta Nishiyama, MD, Kazunori Nakamura, MD, Moritaka Suga, MD, PhD[1], Hidenori Ichiyasu, MD, PhD[6], Takuro Sakagami, MD, PhD[6]

Extended methods

Although this study was a retrospective, single-center study, the data were prospectively collected during the ongoing high-resolution CT (HRCT) study in patients with ARDS. Part of the study data were previously published [2–9]. A total of 210 Japanese patients who were diagnosed as ARDS based on the International definition of ARDS were extracted from our HRCT database constructed from October 1, 2004 to May 31 2017. We also reviewed the data and determined whether or not the individual cases that occurred prior to the publication of the Berlin definition [1] in 2012 met the diagnostic criteria. The prognoses of the individual patients were followed until 180 days after the diagnosis of ARDS. Written informed consent was obtained from all patients or their families. The present study was approved by our institutional review board (Saiseikai Kumamoto Hospital Medical Ethics Committee, Permission number 238), and the study was conducted in accordance with the ethical standards of the Declaration of Helsinki. HRCT scans were performed in all patients on the day of diagnosis of ARDS by their consent. It is important to note that, patients with preexisting chronic interstitial lung diseases were strictly excluded taking the patient disease history, imaging data obtained before the onset of ARDS, and the presence of coarse reticulation and honeycombing on HRCT scans which suggested the existence of chronic pulmonary fibrosis. Furthermore, we did not include patients with other interstitial lung diseases including vasculitis, alveolar haemorrhage or those which were diagnosed as acute organising pneumonia, hypersensitivity pneumonitis or acute eosinophilic pneumonia (Figure 1).

The data of this study was analysed provide historical data to support a randomized, open-label multicentre phase II study to evaluate the efficacy and safety of MultiStem® cells [HLCM051], an allogeneic bone marrow-derived stem cell product, in patients with ARDS

due to pneumonia (NCT03807804), which has been ongoing since February 2019.

Collected data

We collected the clinical data at the diagnosis of ARDS for the patient age, sex, comorbidities, severity of ARDS, McCabe scores, Acute Physiology and Chronic Health Evaluation (APACHE) II scores, sequential organ failure assessment (SOFA) scores, high-resolution CT scores (extent of fibroproliferation), high-resolution CT patterns, disseminated intravascular coagulation (DIC) scores, arterial oxygen tension (PaO_2)/fractional inspired oxygen (FiO_2), the extent of infiltration on chest X-ray and blood test findings. Ventilatory indexes such as PEEP, tidal volume, and peak inspiratory pressure were also collected in the groups (Table 1A, 1B).

Definition of Pneumonia and Method of Microbiological Evaluation

Pneumonia was defined as a new infiltration on chest radiograph at hospital admission with symptoms and signs of lower respiratory infection. If patients were transferred to our hospital from other previous institutions, medical information supplied, including clinical histories and laboratory and radiological data were used for diagnosis.

Sputum, blood, and urine samples, and when available, transbronchial aspirates and bronchoalveolar lavage fluids were obtained for bacterial culture. Nasopharyngeal swabs for respiratory virus detection and urine samples for *S. pneumoniae* and *Legionella pneumophila* were obtained in our emergency department for hospital admission. When any pathogen was not identified in case of prior administration of antibiotics in previous hospitals, the diagnoses were done by medical conferences consisted of more than 10 certificated pulmonologists in our division.

Agreement of sensitivity to the initial empirical antimicrobial agents to detected pathogen in culture

The identification of the detected bacteria or influenza virus antigen as pathogens of pneumonia was determined in conjunction with clinical information. A bacterial pathogen was determined to be present if gram-positive or gram-negative bacteria were detected in a blood sample, sputum, endotracheal aspirates, bronchoalveolar lavage specimen; if *S. pneumoniae* and *Legionella pneumophila* was detected in urine by means of antigen detection. A viral pathogen was determined to be present if influenza virus was detected in a nasopharyngeal swab by means of PCR assay. Except for cases who were immediately diagnosed by urine antigens such as *S. pneumoniae* and *Legionella pneumophila* or by nasopharyngeal swab for flu antigen, antibiotics were empirically used based on the Japanese Respiratory Society guidelines for management of respiratory infections (11,12). When the results of bacterial culture were proven, antibiotics were changed into ones according to the in vitro sensitivity for cultured bacteria. Agreement of sensitivity for initial empirical antimicrobial agents to subsequently cultured pathogens were graded using the following three categories: sensitive, non-sensitive, and no bacteria detected. The category “no bacteria detected” was used when no significant pathogens were cultured, for example when prior administration of antibiotics at the previous institutions were used for the patient.

Assessment of chest radiograph and HRCT findings

Based on the chest radiographs obtained at diagnosis, the extent of bilateral infiltration was classified into the following scores: score 2, half of each unilateral lung field involved: score 3, three-quarters of both lung fields involved: score 4, all of both lung fields involved. All patients underwent helical HRCT scanning of the chest on the day of diagnosis of ARDS using multidetector-row CT (MDCT) scan. All MDCT scans were obtained with 1-mm thickness and 26.5-mm table speed per rotation and were performed

at full inspiration from the lung apex to base. Contiguous CT slices were reconstructed using of a high–spatial frequency algorithm. Sections were displayed at 5-mm intervals throughout the chest with the patient in the supine position and without intravenous contrast medium. No deterioration in the patient's condition during the transfer and examination of CT was seen in any patient. In this study, we evaluated single CT scan acquired on day one of the ARDS diagnosis, because sequential CT scans were hard to be performed after high positive end-expiratory pressure ventilation was introduced. HRCT scans were independently evaluated by two experienced chest radiologists (T.J. and K.F.) who were unaware of the patient condition. The presence and extent of areas of ground-glass attenuation, air-space consolidation, bronchial or bronchiolar dilation, and honeycombing were assessed. Ground-glass attenuation was defined as a hazy area with increased opacification without obscuration of underlying vascular markings. Air-space consolidation was considered present when the vascular markings were obscured. When bronchi were irregular in contour, the dilated bronchus within areas of parenchymal abnormality was recognized as bronchial dilation. Although bronchioles are not observed normally, when branching structures within areas with parenchymal abnormality were seen, the presence of dilated bronchioles was identified. Honeycombing was defined as the presence of cystic airspaces measuring 2-10 mm in diameter with well-defined walls.

Evaluation of HRCT patterns and HRCT score

On HRCT scans, diffuse alveolar damage (DAD) pattern is characterized by patchy ground-glass attenuation and/or air-space consolidation associated with bronchial dilation, reticular opacities, and cystic changes depending on the pathologic fibroproliferative phase of DAD according to the most recent international multidisciplinary consensus statement of idiopathic interstitial pneumonias (13). Three

HRCT patterns (definite DAD pattern, possible DAD pattern, and Inconsistent with DAD pattern) were classified by the modified HRCT patterns of the international guideline for idiopathic pulmonary fibrosis in each patient on the basis of HRCT findings (see Supplemented Figure 1). According to this guideline, “definite” DAD pattern on HRCT scans was defined as bilateral patchy ground-glass attenuation and/or air-space consolidation with bronchial dilation. Possible DAD pattern was identified when bilateral ground-glass attenuation and/or air-space consolidation was not associated with bronchial dilation on HRCT scans. Inconsistent with DAD pattern was defined as bilateral segmental or lobar air-space consolidation with/without centrilobular opacities. HRCT findings were graded as1-6 based on the following classification system correlating with the previously described pathology (14): 1, normal attenuation; 2, ground-glass attenuation; 3, consolidation; 4, ground-glass attenuation with traction bronchiolectasis or bronchiectasis; 5, consolidation with traction bronchiolectasis or bronchiectasis; 6, honeycombing. The presence of each of these six abnormalities was assessed independently in three (upper, middle, lower) zones of each lung. The upper zone was defined as the area above the level of the carina, the middle zone as the area between the level of the carina and that of the infrapulmonary vein, and the lower zone as the area below the level of the infrapulmonary vein. The extent of each abnormality was determined by visually estimating the percentage (to the nearest 10%) of the affected lung parenchyma in each zone. The assessments of the two observers were averaged. The abnormality score for each zone was calculated by multiplying the percentage area by the point value (1-6). The six zone scores for the individual patients were averaged to determine the total score for each abnormality. The overall CT score for each patient was obtained by

adding the six averaged scores. The scoring system was previously reported (2,3,14).

Treatment

Ventilatory management was implemented according to the evidence-based guidelines, with reference to the lower tidal volume (V_T) based on predicted body weight (PBW) (6 < V_T < 10 ml/kg PBW) in the ARDS Clinical Trial (15). Weaning and discontinuing ventilatory support was referenced to the evidenced-based guidelines of the American College of Chest Physicians (16). The plateau pressure was limited to < 30 cmH_2O with a positive end-expiratory pressure (PEEP) of 8-12 cmH_2O. PEEP, peak inspiratory pressure (PIP), and V_T were recorded daily. One patient with influenza virus induced ARDS and another patient with ARDS due to severe *Streptococcus pneumonia* infection were treated with extracorporeal membrane oxygenation.

Screening of ventilator-associated outcomes

We reported that early fibroproliferation on HRCT scans at diagnosis of ARDS increased risk of prolonged ventilation, ventilator-associated pneumonia, and air leak syndrome, resulting in secondary sepsis syndrome (3). To evaluate the relation between the HRCT scores and these ventilator-associated outcomes in patients with ARDS due to pneumonia, these outcomes were recorded; whether the patient was weaned from the ventilator within 28 days after the diagnosis; whether air leak syndrome defined as any pneumothorax, pneumomediastinum or subcutaneous emphysema was noted as present or absent on regular chest radiographs; whether diagnoses of culture-confirmed ventilator-associated tracheobronchitis or pneumonia requiring newly prescribed antibiotics were done.

Evaluation of Coagulative and Fibrinolytic Abnormalities

Coagulative and fibrinolytic abnormalities at diagnosis of ARDS were assessed by the DIC score in accordance with the diagnostic criteria by the Japanese Association of Acute

Medicine (JAAM) (17)

Statistical analysis

Continuous variables expressed as medians and interquartile ranges (IQRs), and categorical variables in each three group (survived, early death and late death) are shown. The interobserver variation of the presence/absence of the HRCT findings or overall patterns was analysed using the weighted *kappa* statistic. Interobserver agreement was classified as follows: poor (*kappa* =0-0.20), fair (*kappa* =0.21-0.40), moderate (*kappa* =0.41-0.60), substantial (*kappa* =0.61-0.80), and almost perfect (*kappa* =0.81-1.00). The interobserver variation of the extent of the HRCT findings was assessed by Spearman's rank correlation coefficient. The HRCT scores of two independent observers' measurements were compared with the Bland-Altman method. If the HRCT patterns or the HRCT scores did not agree between the two radiologists, one of the patterns or the scores was adopted by consensus. Of the HRCT scores which were semiquantitative markers of fibroproliferation, and the three patterns on HRCT scan (definite DAD pattern, possible DAD pattern, and inconsistent with DAD pattern), which were qualitative indicators, the HRCT scores were adopted for multivariate analysis because of the results of our previous studies (2-9). From our published studies (2-9) as described in our study hypothesis, we built the model for multivariate analysis using age, the APACHE II score, the HRCT score, and the DIC score.

To evaluate whether the APACHE II score would be a prognostic factor of early death (< 7-day mortality), univariate and multivariate analyses using Cox proportional hazard models were performed among the two groups; early death group vs. late death group plus survived one. Similarly, to assess whether the HRCT score would be predictive factors for the late death (from day 7 to day 180), univariate and multivariate analyses

were done between the two groups; late death group vs. survived one. Using ROC curve, we examined the sensitivity, specificity and predictive values of the APACHE II score and the HRCT score and identified the best cut-off value of each by using Youden's index. For all statistical analyses, a P value of less than 0.05 was considered to indicate a statistically significant difference. We used the EZR software (Saitama Medical center, Jichi Medical University, Saitama, Japan), which is a graphical user interface for R VV.3.2.2 (The R Foundation for Statistical Computing, Vienna, Austria), for all statistical analyses and SPSS software (V.22.0) for the factor analysis.

Extended Methods Reference

1. ARDS Definition Task Force, Ranieri VM, Rubenfeld GD, et al. Acute respiratory distress syndrome: The Berlin definition. *JAMA* 2012;307:2526–33
2. Ichikado K, Suga M, Muranaka H, et al. Prediction of prognosis for acute respiratory distress syndrome with thin-section CT: validation in 44 cases. *Radiology* 2006;238:321–9.
3. Ichikado K, Muranaka H, Gushima Y, et al. Fibroproliferative changes on high-resolution CT in the acute respiratory distress syndrome predict mortality and ventilator dependency: a prospective observational cohort study. *BMJ Open* 2012;2:e000545.
4. Kawamura K, Ichikado K, Takaki M, et al. Efficacy of azithromycin in sepsis-associated acute respiratory distress syndrome: a retrospective study and propensity score analysis. Springerplus. 2016 Jul 28;5(1):1193. doi: 10.1186/s40064-016-2866-1. eCollection 2016
5. Takaki M, Ichikado K, Kawamura K, Gushima Y, Suga M. The negative effect of initial high-dose methylprednisolone and tapering regimen for acute respiratory distress syndrome: a retrospective propensity matched cohort study. Crit Care. 2017 Jun 8;21(1):135. doi: 10.1186/s13054-017-1723-0.
6. Anan K, Ichikado K, Kawamura K, Johkoh T, Fujimoto K, Suga M. Clinical characteristics and prognosis of drug-associated acute respiratory distress syndrome compared with non-drug-associated acute respiratory distress syndrome: a single-centre retrospective study in Japan. BMJ Open. 2017 Nov 8;7(11):e015330. doi: 10.1136/bmjopen-2016-015330.
7. Kawamura K, Ichikado K, Takaki M, Eguchi Y, Anan K, Suga M. Adjunctive therapy with azithromycin for moderate and severe acute respiratory distress syndrome: a retrospective, propensity score-matching analysis of prospectively collected data at a single center. Int J Antimicrob Agents. 2018 Jun;51(6):918-924.
8. Anan K, Kawamura K, Suga M, Ichikado K. Clinical difference between pulmonary and extrapulmonary acute respiratory distress syndrome: a retrospective cohort study of prospectively collected data in Japan. J Thorac Dis 2018 Oct;10(10):5796-5803.
9. Anan K, Ichikado K, Ishihara T, et al. A scoring system with high-resolution computed tomography to predict drug-associated acute respiratory distress

syndrome: development and internal validation. Sci Rep 2019 Jun 13;9(1):8601. doi: 10.1038/s41598-019-45063-9.

10. Cilloniz C, Ferrer M, Liapikou A et al. Acute respiratory distress syndrome in mechanically ventilated patients with community-acquired pneumonia. Eur Respir J 2018 51: 1702215; **DOI:** 10.1183/13993003.02215-2017
11. Yanagihara K, Kohno S, Matsushima T. Japanese Guidelines for the management of community-acquired pneumonia. Int J Antimicrob Agents. 2001;18:Suppl 1:S45-48.
12. Miyashita N, Matsushima T, Oka M, and Japanese Respiratory Society, The JRS guidelines for the management of community-acquired pneumonia in adults: an update and new recommendations. Intern Med 2006;45(7):419-428.
13. Travis WD, Costabel U, Hansell DM, et al. An Official American Thoracic Society/European Respiratory Society Statement: Update of the International Multidisciplinary Classification of the Idiopathic Interstitial Pneumonias. Am J Respir Crit Care Med 2013;188(6):733-748
14. Ichikado K, Suga M, Müller NL, et al. Acute interstitial pneumonia: comparison of high-resolution computed tomography findings between survivors and nonsurvivors. Am J Respir Crit Care Med. 2002 Jun 1;165(11):1551-6..
15. Brower RG, Matthay MA, Morris A, et al. Ventilation 22. with lower tidal volumes as compared with traditional tidal volumes for acute lung injury and the acute respiratory distress syndrome. N Engl J Med 2000;342:1301-8.
16. MacIntyre NR, Cook DJ, Ely EW Jr, et al. Evidence- based guidelines for weaning and discontinuing ventilatory support: a collective task force facilitated by the American College of Chest Physicians; the American Association for Respiratory Care; and the American College of Critical Care Medicine. Chest 2001;120:375-95S.
17. Gando S, Iba T, Eguchi Y, et al. A multicenter, prospective validation of disseminated intravascular coagulation diagnostic criteria for critically ill patients: comparing current criteria. Crit Care Med 2006;34:625-31.

Supplementary Figure Legends

Supplementary Figure 1. Receiver operator characteristic (ROC) curve of the predictive value of the APACHE II score for early death (< 7 days from diagnosis)

The ROC curve identified the optimal cutoff value of 27 determined by the Youden Index for the prediction of death within 6 days, with 67% sensitivity and 84% specificity (AUC, 0.79; 95%CI, 0.68-0.91).

Supplementary Figure 2. Classification of high-resolution CT patterns

Definite diffuse alveolar damage pattern

The HRCT pattern consists of bilateral arears of increased attenuation (ground-glass attenuation or consolidation) with traction bronchiectasis (arrows).

Possible diffuse alveolar damage pattern

The HRCT pattern is characterized by bilateral areas of increased attenuation (ground-glass attenuation or consolidation) without traction bronchiectasis.

Inconsistent with diffuse alveolar damage pattern

The HRCT pattern consists of lobar or segmental areas of consolidation with/without ground-glass attenuation.

Supplementary Figure 3. Bland-Altman plots
